# Linking Findings in Microfluidics to Membrane Emulsification Process Design: The Importance of Wettability and Component Interactions with Interfaces

**DOI:** 10.3390/membranes6020026

**Published:** 2016-05-11

**Authors:** Karin Schroën, Montse Ferrando, Silvia de Lamo-Castellví, Sami Sahin, Carme Güell

**Affiliations:** 1 Food Process Engineering Group, Department of Agrotechnology & Food Science, Wageningen University, Bornse Weilanden 9, Wageningen 6708 WG, The Netherlands; sami.sahin@wur.nl; 2 Departament d’Enginyeria Química, Escola Tècnica Superior d’Enginyeria Química, Universitat Rovira i Virgili, Avda. Països Catalans 26, Tarragona 43007, Spain; montse.ferrando@urv.cat (M.F.); silvia.delamo@urv.cat (S.L.-C.); carme.guell@urv.cat (C.G.)

**Keywords:** microfluidics, emulsification, wettability changes, contact angle, process stability

## Abstract

In microfluidics and other microstructured devices, wettability changes, as a result of component interactions with the solid wall, can have dramatic effects. In emulsion separation and emulsification applications, the desired behavior can even be completely lost. Wettability changes also occur in one phase systems, but the effect is much more far-reaching when using two-phase systems. For microfluidic emulsification devices, this can be elegantly demonstrated and quantified for EDGE (Edge-base Droplet GEneration) devices that have a specific behavior that allows us to distinguish between surfactant and liquid interactions with the solid surface. Based on these findings, design rules can be defined for emulsification with any micro-structured emulsification device, such as direct and premix membrane emulsification. In general, it can be concluded that mostly surface interactions increase the contact angle toward 90°, either through the surfactant, or the oil that is used. This leads to poor process stability, and very limited pressure ranges at which small droplets can be made in microfluidic systems, and cross-flow membrane emulsification. In a limited number of cases, surface interactions can also lead to lower contact angles, thereby increasing the operational stability. This paper concludes with a guideline that can be used to come to the appropriate combination of membrane construction material (or any micro-structured device), surfactants and liquids, in combination with process conditions.

## 1. Introduction

For emulsification, many micro-structured devices have been used such as membranes, and various microfluidic devices, but even the nozzles of high pressure homogenizers may qualify as such (for recent extensive reviews, see, e.g., [1,2]). Various process parameters have been investigated and scaling relations have been derived [3], and up-scaling has also been considered [4].

It should be mentioned that there is a lot of contradicting information in the literature. In some cases, it is reported that a certain emulsion can be made very efficiently with a specific membrane, while other authors that use seemingly the same combination are not able to make the emulsion, or only for a very short time [5]. Besides, there are considerable differences between the modes of operation; in some cases, cross-flow emulsification works well, while pre-mix emulsification does not work at all while trying to make the same emulsion with the same membrane; specific examples will be given in the respective sections.

We think that these discrepancies can be explained by the fact that the emulsion components interact with surfaces and interfaces (both liquid and solid), since this leads to the energetically most favorable option for the system as a whole. Because emulsification involves the creation of a lot of interface, and through that an increase in the Gibbs free energy, the surface active components will reduce the interfacial tension and therewith also the Gibbs free energy.

(1) ΔG=σdA

where Δ*G* is the Gibbs free energy, σ is the interfacial tension, and is *dA* the increase in interfacial area. This is a well-known fact, and has been discussed in many papers, also in relation to micro-structured devices, including dynamic interfacial tension effects [6,7,8]. A good overview of surfactant effects in microfluidics was performed by Baret [9].

Besides, there is another interface that plays a big role in emulsification, and that is the solid interface. What is normally postulated is that you need a membrane/microfluidic device that is non-wetting for the to-be-dispersed phase to prepare an emulsion with a certain continuous phase (e.g., [10,11,12,13,14,15]). In cross-flow membrane emulsification and microfluidic devices this implies that a hydrophilic (membrane) surface is needed to make an oil-in-water emulsion and a hydrophobic (membrane) surface for water in oil emulsions. In premix emulsification, a hydrophilic surface is used to refine an oil-in-water emulsion, and a hydrophobic one for water in oil emulsification [16]. In some rare cases, a very concentrated premix emulsion can be phase reversed by pushing it through a membrane with polarity equal to the dispersed phase [17].

These rules of thumb may seem to be very easy to use, but what is mostly not considered is that if a combination of, e.g., hydrophilic surface/membrane and hydrophobic oil is used, this also implies an effect on the Gibbs free energy. The internal area of any micro-structured device can be very large, and putting this in contact with a non-wetting phase implies a large increase in Gibbs free energy. The system as a whole will strive for a reduction of the Gibbs free energy, and this also implies that there is a driving force for either surfactant-solid interface, or liquid-solid interactions. This may change the wettability of a system completely as demonstrated in experimental work by, e.g., Keurentjes *et al*. [18] for emulsion separation, and Hüsken *et al*. [19] for two-phase membrane bioreactors. Further, in simulations, the effect of a change in contact angle on emulsification with microsieves has been demonstrated to result in sticking of the to-be-dispersed phase, thereby leading to loss of droplet formation [20].

**Figure 1:**
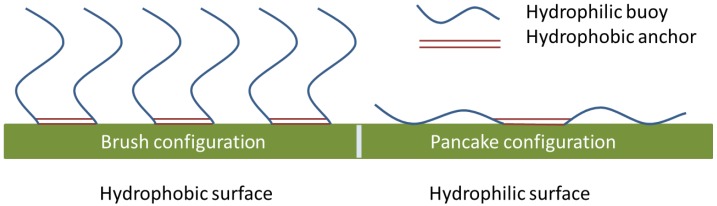
Schematic representation of the adsorption of a polyethylene oxide-polypropylene oxide-polyethylene oxide block co-polymer onto a hydrophobic and hydrophilic surface. Only in the brush configuration protein adsorption is prevented, and the wettability of the surface remains intact.

An illustrative example on the rigorous effects of wettability changes on process operation can be found in the early work of one of the authors on emulsion separation with membranes. The emulsion contained the enzyme lipase that was used to hydrolyze vegetable oil into free fatty acids, and the emulsion was separated with a hydrophobic polypropylene membrane [21]. When starting the experiment, the flux immediately decreased very rapidly due to binding of lipase that is able to form very strong interactions with any hydrophobic interface. Besides, the pressure that could be used to separate the emulsion decreased very rapidly; in first instance, only the oil phase permeated, but within a day water also permeated the membrane at very low pressure, indicating that the wettability of the membrane had changed drastically (due to adsorption and diffusion of lipase into the membrane pores [22]). Only when the membrane was modified with a specific block copolymer that prevented lipase adsorption [23], while still keeping its wettability [24], the membrane could be operated at high pressure, and for long periods of time (tests of up to six weeks were performed). The underlying mechanism is schematically represented in Figure 1; in the brush configuration, the surface has protein repelling properties due to the presence of the long hydrophilic polyethylene oxide buoy groups, while it is anchored by the hydrophobic polypropylene oxide group, which allowed retention of the hydrophobic nature of the membrane. If adsorbed onto a hydrophilic surface, the block co-polymer assumes a pancake configuration that is not protein repelling.

This effect has not only been demonstrated with enzymes, but also with two phase systems used for down-stream processing of microbial bioconversions [19,25], and for surfactant containing emulsions [18]. In one phase systems these effects are not that relevant; e.g., in membrane separation this would be termed fouling, or flux loss, but the process as such would not be lost because of component surface interactions.

This clearly indicates the importance of surfactant interactions, but in membrane and microfluidic emulsification they are not often taken as a starting point for process design. It could be that if a combination does not work, it can also not be found in literature because people have moved on to try other options. We will report on membrane emulsification, both cross-flow (or direct) and premix emulsification, and specifically compare with the spontaneous droplet formation technique called EDGE (Edge-based Droplet Generation; [26,27,28]). This technique was chosen, for its large viewing area in which wettability changes can be observed. Besides, the pressure dependency of droplet formation allows charting wettability issues, as will be explained in the next section, that starts with a general introduction of the EDGE technology.

It is good to mention that there are other microfluidic devices that use spontaneous droplet formation, and for which wettability effects are relevant. For microchannel emulsification that was proposed by Kawakatsu *et al.* [29], and its’ up-scaled relative straight through microchannels [30,31,32], the droplet formation mechanism was described by Sigiura and coworkers [33], and various comments were made related to wettability effects [34,35,36,37]. Besides, in the system proposed by Dangla *et al*. [38], in which a tapered droplet formation unit is used, wettability plays an important role.

## 2. EDGE Devices

### 2.1. General Behavior

**Figure 2:**
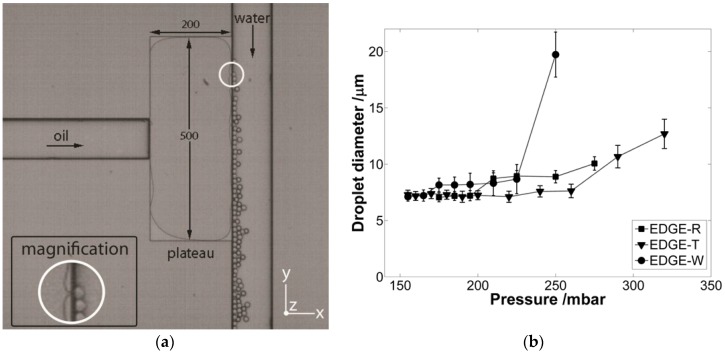
(**a**) Top view of an EDGE device; the oil is pushed from the left onto the plateau, which is a shallow area of two micrometer. Upon reaching the deeper channel on the right, the oil droplets are generated over the entire length of the plateau [39]; (**b**) pressure dependency of EDGE devices; droplet formation starts at the minimum pressure, and the droplet size remains constant till the blow-up point is reached at which the droplet size shoots up, and the droplets become polydisperse [40].

The EDGE device is schematically shown in Figure 2, together with its specific pressure dependency profile. The to-be-dispersed phase is pressurized onto the plateau, and the minimum pressure at which this can take place corresponds to the Laplace pressure:

(2) $$\Delta P_{min}=\sigma \left(\frac{1}{R_{1}}+\frac{1}{R_{2}}\right)\cos\theta$$

where Δ*P_min_* is the minimum invasion pressure, σ is the interfacial tension, *R*_1_ and *R*_2_ are the height and width of the plateau, respectively, and θ is the continuous phase contact angle [37]. Since the height of the plateau is much smaller than the width, the curvature related to the width cancels out. In this equation, both the effects of components on the liquid-liquid interface (σ), and at the solid liquid interface (θ) are visible [26].

At pressures above the minimum pressure, the plateau fills up, as can be seen in Figure 2, and droplet formation starts. Mostly, upon increasing pressure the droplet size remains fairly constant; the flow of liquid on the terrace is such that droplets can leap into the deeper channel while still connected, and upon reaching a certain size they detach. This situation can be described by the so-called flux criterion derived by van Dijke and coworkers for microchannels [38]. In these systems, the flow toward the neck that keeps the droplet connected can be defined as:

(3) $$\phi_{p,n\;}=\;\frac{\pi R^{4}}{8\eta L}\left(P_{app}-\frac{\sigma \cos\theta }{R_{2}}\;\right)$$

where Φ*_p,n_* is the liquid flow from the plateau toward the neck, and π*R*^4^/8η*L* relates to the Poisseuille flow, and pressure difference between the applied pressure (*P_app_*) and that in the neck (see also Equation (2)). Besides, there is a flow from the neck to the droplet, which is defined as:

(4) $$\phi_{p,n\;}=\;\frac{\pi R^{4}}{8\eta L}\left(\frac{\sigma \cos\theta }{R_{2}}-\;\frac{2\sigma }{R_{drop}}\right)$$

where the Laplace pressure in the drop is 2σ/*R_drop_*.

As long as the flow toward the neck does not exceed that from the neck to the droplet, the droplet will remain attached until it reaches a certain size at which the flow to the droplet exceeds the supply, and the droplet is released. This situation mostly occurs for a certain pressure range. At even higher pressures, the neck can no longer collapse due to the high supply flow and blow-up occurs as also indicated in Figure 2. It is clear that depending on the liquid/liquid (σ) and liquid/solid (θ) interactions, droplet formation will change, as will be discussed in greater detail in the next section. From Figure 2, it is also clear that the specific design of the EDGE (T corresponds to a more triangular, and W to a very wide design) does influence its behavior [40], but we will keep that as much as possible out of the considerations presented here and focus on one specific design, the design in Figure 2.

In the next sections, we first describe the effect of contact angle on pressure stability [41], after which a section is presented in which wettability is influenced by surfactant and oil interactions with the construction material. 

### 2.2. Contact Angle

**Figure 3:**
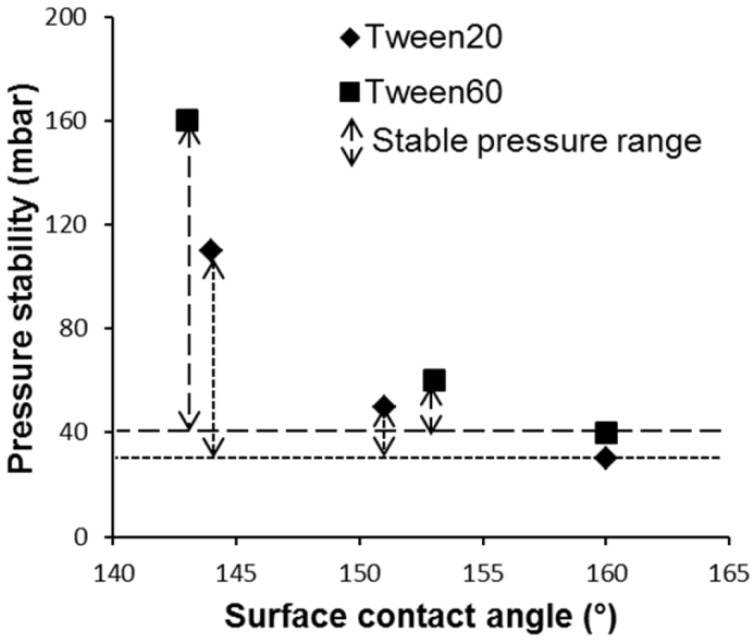
Pressure stability ranges of EDGE devices modified with silanes. The three phase contact angles in the presence of Tween 20 or 60 are given on the *x*-axis, and it is clear that the operational stability for direct emulsification is a strong function of the contact angle/surface wettability [41].

In the work of Maan *et al*. [41], the effect of contact angle was evaluated by modification of the surface with different silanes. The three phase contact angle was measured in the presence of Tween 20 and Tween 60, and the operational pressure stability was recorded, as shown in Figure 3.

From Figure 3 it can be concluded that the operational stability is a very strong function of the contact angle, which was expected to be the case. The minimum pressure at which the to-be-dispersed liquid can replace the continuous phase is low (Equation (2)), and also the pressure in the neck is low, and this leads to a situation in which the flux criterion (Equations (3) and (4)) is exceeded already at low pressure. For both surfactants, this behavior was found; please note that for the conditions that were used, these effects are the result of surfactant interactions with the liquid/liquid interface, not with the solid surface.

### 2.3. Component Surface Interactions

#### 2.3.1. Concentration Effects

It is actually very rare to have no surfactant surface interactions that influence the contact angle; the previous section is more an exception than a rule. Both for regular EDGE devices [42], and EDGE with metal parts [43,44], it was observed that these interactions greatly influence pressure stability of the devices, negatively and positively. For the devices with metal inserts, the interested reader is referred through to Maan *et al*. [43] for oil in water emulsions in which the effect of water soluble emulsifiers is discussed, and [44] for water in oil emulsions and oil soluble substances. The effects described in these two papers were also found in regular EDGE devices and those are discussed in the section on various components.

**Figure 4:**
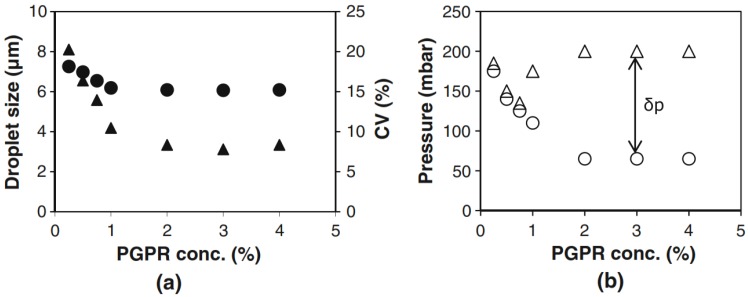
Droplet size (filled circles) and coefficient of variation (filled triangles) as function of the PGPR concentration used (**a**); Minimal pressure for droplet formation (open circles); and blow-up pressure (open triangles as function of PGPR concentration (**b**) [44].

We first present the effect of the concentration of the emulsifier on pressure stability as shown in Figure 4 [43]. At low PGPR concentration, the droplet size is larger than expected for a one-micrometer high plateau (the scaling ratio between droplet size and plateau height is 6), and also the coefficient of variation is rather high for an EDGE system as shown in Figure 4a. The low concentrations also correspond to very low pressure ranges, as indicated in Figure 4b. Especially at low concentration, the entrance pressure is high, and this has to do with the higher interfacial tension at lower concentration (Equation (1)), and possibly also to a different contact angle, which leads to higher Laplace pressures. Because of this, the blow-up pressure is very close to the minimal pressure, and this leads to very narrow operating conditions, as shown in Figure 4b.

At higher surfactant concentrations, the droplets are much more monodisperse, their size is according to the scaling relation, and the pressure range is much wider. From this, it is also clear that a certain amount of surfactant is needed to get the system to work. In the current paper, we only relate this to the formation of the droplets not to stability of the droplets after production. This latter effect is another optimization question, although it should be mentioned that the droplets that are produced with EDGE are mostly very stable. At higher concentrations, it is expected that the liquid/liquid interface is at its equilibrium interfacial tension, which only decreases very weakly at concentrations >1%. It is also expected that the device interface is completely at equilibrium with the continuous phase. This all leads to a constant contact angle, which would result in constant blow-up pressure, droplet size and low coefficient of variation as shown in Figure 4a [44]. Although suggested in literature, it is unlikely that mass transfer/diffusion limitations could explain these effects.

#### 2.3.2. Various Components

In the previous section, PGPR was discussed, but also various other components were tested in EDGE devices, and the most concise comparison can be found in Sahin and Schroën [5], who systematically varied surface active components that were always used at such concentrations that they correspond to the plateau region in Figure 4. Besides, the emulsification behavior of hexadecane and sunflower oil were reported, as shown in Table 1. It is needless to say that there is a lot of information in Table 1; but we will try to summarize those results that we find most illustrative for the effects that occur, and of most relevance to membrane emulsification experiments.

Away from the difference in frequency that is caused by, amongst others, the difference in viscosity of the two oils (factor of 15–20), it is immediately clear that the pressure ranges for both oils can be very different, also when using the same emulsifier. In general, hexadecane does not have a strong interaction with the glass surfaces of the microfluidic devices, which also leads to low continuous phase contact angles and that together with the higher interfacial tension compared to sunflower oil leads to higher entrance pressures (see Equation (2)).

For SDS that does not have a very strong interaction with glass, there is a remarkable difference between the two oils that are used. Sunflower oil only has a very narrow pressure range (20 mbar), while hexadecane has a much wider range (50 mbar), and that is due to the fact that sunflower oil can interact with the surface and displace SDS [45], possibly forming thin films that render the surface hydrophobic. When first treated with whey protein isolate that mostly consists of β-lactoglobulin (that is known for its strong interaction with the surface; second entry in the table), the pressure range gets much wider. This indicates that it is actually a hydrophilic protein layer that serves as the surface from which the droplets are released. This is in line with findings of van Dijke and coworkers [26], who investigated food emulsions made with EDGE devices, and who succeeded in making emulsions with sunflower oil, and even double emulsions, but could not use sunflower oil in combination with SDS. Compared to SDS, Tween 20 has a much stronger interaction with the glass surface, and is not displaced by the sunflower oil, and with both oils good pressure stabilities were found.

For practically all proteins mentioned in the table, the pressure ranges that were found are wide for situations in which protein interaction with the glass surface is favorable, as is reflected in the results obtained at different pH. When investigating BSA stabilized emulsions at high pH, BSA and glass have negative charge [46], leading to nice pressure ranges. At low pH, where the glass has hardly any charge, some interactions may still occur through specific groups in the protein; however, the interaction is expected to be much less pronounced as at high pH. When using hexadecane, the binding strength of the protein is apparently still sufficient to warrant emulsification, while for sunflower oil emulsification was lost, most probably because of strong interaction of oil and surface, leading to replacement of the protein and possibly oil film formation. At its iso-electric point, BSA shows large droplets both for hexadecane and sunflower oil at a rather wide pressure range (70 and 200 mbar for sunflower oil and hexadecane respectively), which is not completely understood. It could be that since the protein has no net charge, it forms small protein aggregates (these were found to occur) that could stabilize the emulsion through a Pickering mechanism [47].

Also in the group of Kobayashi [48], and in the work of Saito and coworkers [49,50], the effect of pH was investigated for similar protein, SDS and Tween stabilized emulsions but now using straight-through micro-channels. The authors suggest that the charge of the droplets and the interface “affects the smoothness of droplet formation and the behavior of the droplets formed”. Although we agree that charges can have an effect, in our view, wettability changes due to the use of different pH and salt should also be considered, and could have an even greater effect than charges.

#### 2.3.3. Surface Roughness

To be complete, the effect of roughness on operational pressure stability of the EDGE plateau is discussed for semi-metal devices that had Copper or Copper-Nickel inserts [43,44]. On the plateaus, so-called fingering behavior was observed that was linked to the roughness of the plateau, and which limited the amount of droplet formation points, but depending on the roughness the pressure ranges were also much wider. Both effects counterbalanced, and ultimately the productivity of metal EDGEs was approximately the same as found for the regular EDGE devices. When re-structuring the plateaus into many small plateaus as done in the work of Sahin and Schroën [51], it is possible to increase the productivity considerably; both the number of droplet formation points and the pressure stability are in that case increased.

To summarize, from the investigations done with EDGE devices, favorable combinations of components, construction materials, and process conditions can be identified, and it is expected that these also hold for other micro-structured devices, such as T-junctions, and membranes. Both will be discussed together since they emulate the same conditions. Besides, pre-mix emulsification is discussed together with the very limited information that is available on microfluidic studies.

## 3. Membrane Emulsification

Contrary to EDGE emulsification in which spontaneous droplet formation takes place, in membrane emulsification shear based droplet formation occurs. For overviews on shear based membrane emulsification, please consult the work of Joscelyne and Trägårdh ([52] review food applications), Charcosset and coworkers ([53] general review), Vladisavljevic and Williams ([54] general overview with many products), Lambrich and Schubert ([55] microstructured devices), Spyropoulos *et al*. ([56,57] general reviews), van der Graaf and coworkers ([58] double emulsions), Charcosset ([11] specific for food), and Nazir *et al*. ([16] premix emulsification). The patent literature was reviewed by Piacentini *et al*. [59].

**Figure 5:**
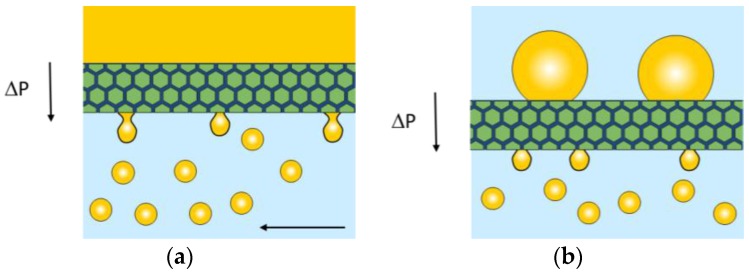
Schematic representations of cross-flow emulsification with the liquid pressurized through the membrane, and the droplets sheared of by the cross-flowing continuous phase (**a**); and premix emulsification in which a coarse emulsion is pressurized through the membrane, leading to smaller droplets (**b**).

We will focus on the classic shear based systems in which droplets are made by pushing a liquid through a membrane pore or tiny channel into a cross-flowing other liquid. Besides, premix emulsification is discussed. Both processes are schematically shown in Figure 5. Alternative shearing methods have been described in literature such as rotation [60,61], vibration and other dynamic effects, and these techniques are handsomely reviewed by Jaffrin [62].

### 3.1. Cross-Flow or Direct Droplet Formation

As schematically indicated in Figure 5a, during cross-flow emulsification the to-be-dispersed phase is pushed through a membrane. The liquid that emerges from the membrane surface is sheared of as droplets. Cross-flow emulsification has been investigated extensively; the most popular membranes for oil in water emulsions are made of Shirasu porous glass (SPG) that was discovered by Nakashima and Shimizu [63,64], and ceramic materials such as alumina oxide (e.g., [15]). Besides, work was done using α-alumina- and zirconia-coated membranes [64], macro-porous silica glass membranes [65,66], and micro-fabricated metal membranes [67,68]. A special category are the microsieves, which are microfabricated devices with extremely uniform pores covered with a thin silicon nitride layer [69], that have been investigated by e.g., Geerken *et al*. [70], Abrahamse *et al*. [20,71] and Zhu and Barrow [72]. A comparison in relation to process design can be found in Gijsbertsen Abramhamse *et al*. [73].

For water in oil emulsification, PTFE (polytetrafluoroethylene) membranes, and hydrophobized SPG membranes have been used [17,74,75,76]. To be complete, it should be mentioned that micro-fabricated metal membranes [77] and hydrophobized silicon nitride microsieves [78] have also been successfully applied, thereby indicating the relevance of wettability by the continuous phase.

Based on the wettability requirement described earlier, it can be expected that the contact angle plays a role in the droplet formation mechanism. However, early investigations on droplet formation with membranes (e.g., [15,78]) have identified mechanisms that are based on force balances (critical capillary number, which is independent of contact angle). Further, droplet formation has been investigated, through experimentation with microfluidic devices (e.g., [79,80,81]), and simulations (e.g., [82,83], as recently reviewed by Muijlwijk and colleagues [84]). Only in simulations, the contact angle can be varied systematically, and this was done in the simulations of Abrahamse and coworkers [20], who found large effects on droplet release; thereby, clearly signifying its importance. Quantifying this effect in an experimental setting is far from trivial, and in the process guidelines section, we suggest how these effects can be assessed.

It was reported that SPG membranes can make droplets in the absence of cross-flow (e.g., [85]), using a droplet formation mechanism similar to that of microchannels (introduced in 1997 by Kawakatsu and coworkers [29]). These membrane are rather thick, and the porous structure is well connected [86], which allows for local continuous phase intrusion, leading to uniform droplets (e.g., Sugiura *et al*. [35,36]). For this scenario, wettability by the continuous phase is also a requirement.

### 3.2. Premix Droplet Formation

In Figure 5b, premix emulsification is schematically depicted, with the coarse premix being pushed through the membrane from the top, which leads to emulsion refinement upon passing the membrane. The technique was introduced by Suzuki *et al*. [17], and as mentioned previously, mostly a membrane is used that is wetted by the continuous phase, and for oil in water emulsions SPG membranes are mostly used as was recently reviewed by Nazir and coworkers [16]. Also α-alumina, polycarbonate, polyamide and cellulose have been reported, together with glass filters for oil in water emulsions, and for water in oil emulsions, PTFE, PE, and hydrophobized SPG membranes were applied. Besides single emulsions, more complex structures were reported to be produced successfully, including double emulsions, polymeric beads, capsules, *etc.* (reviewed in [16]). As mentioned previously, if the membrane is wetted by the dispersed phase, phase inversion can take place, leading to very high disperse phase volume fractions [17].

The mechanism of droplet formation during premix emulsification is hardly investigated. Van der Zwan and coworkers [87] used a microfluidic device and mentioned that many different droplet mechanisms operate at the same time during pre-mix emulsification, ranging from snap-off due to localized shear forces, break-up due to interfacial tension effects (Rayleigh and Laplace instabilities), and break-up due to steric hindrance between droplets. Surprisingly enough, the droplet size distributions are narrow and in the same range as the pore size distributions for SPG membranes [1].

### 3.3. Comparison with Literature

In the review of Nazir and coworkers [16], a list is given of the various combination of emulsifiers and oils that have been reported to work for membrane emulsification. The list comprises both cross-flow and premix emulsification, and oil in water (O/W) and water in oil (W/O) emulsions, and some related products. When taking the findings with EDGE as a starting point, it is expected that when targeting oil in water emulsions with SPG membranes (or any other glass surface), the combination of alkane and SDS, Tween, or protein would work. When using a vegetable oil, it is expected that SDS would not work but the other emulsifiers would work. For water in oil emulsification it is expected that combinations with PGPR would work.

Of the 30 entries in the table that relate to emulsions, only two report that SDS and vegetable oil was used [85,88]. In the work of Kukizaki and Goto [88], a new SPG membrane is presented with a rather complex structure that deviates from the standard SPG membrane, and also contains alumina oxide. If this last material dominates emulsification, the result can be understood since the interaction of SDS with this ceramic material is much better than with glass. It should also be mentioned that the pore activation is very low, 2%–4%, and this could also be indicative for adverse wettability effects taking place; normally the porosity of SPG membranes is very high, as was reported by Vladisavljevic and coworkers [86]. In the study of Yasuno and colleagues [85], in which actually no cross-flow was applied and spontaneous emulsification was allowed to take place, very low pore activation (0.3%–0.5%) was found, which could very well be caused by wettability changes that prevent inflow of the continuous phase into the membrane.

When focusing on polymeric membranes, it is even clearer how membrane properties influence the emulsification process. Polycarbonate membranes could not be successfully employed to produce stable emulsions with Tween 20 or BSA [89] regardless of the pressure applied, and most probably this is caused by the hydrophobic nature of the construction material. An alternative explanation is that the membrane is very thin, and that could induce less droplet break-up than thicker membranes.

For polyamide, polyethersulfone or nitrocellulose mixed ester membranes, with thicknesses between 100 and 200 μm, the ability to produce a stable emulsion depended largely on the type of emulsifier (see also Table 2). When using 0.8 μm polyethersulfone (PES) and a nitrocellulose mixed ester (MCE) membranes with different surface porosity and thickness, the droplet size (*d*_3,2_) was about 1 μm when using Tween 20. When using BSA, the cross-flow velocity had to be increased, while the final droplet size was three to five times the pore diameter depending on the BSA concentration and membrane used (Table 2). The equilibrium interfacial tension cannot solely explain the observed differences, as can be seen in Table 2 where the interfacial tension values and the droplet size at the end of emulsification are presented. Through FTIR analysis performed on membranes [90] used for emulsification with BSA, it was found that most of the surface fouling was oil, which indicates that sunflower oil replaces BSA at the surface, and a film is formed that hampers droplet formation [45]. Besides, the ability of the protein to stabilize the oil-water interface will also depend on the protein maintaining its functionality, since if it is lost, oil droplets will coalesce and deposit on the hydrophilic membrane surface, hindering the emulsification process, which is in line with the findings reported for EDGE emulsification.

Alternatively, also complexes can be considered as stabilizing agent; soluble whey protein-polysaccharide complexes can stabilize the O/W and the W/O interface of single and double emulsions, respectively [92,93,94]. These more complex interfacial structures did not affect the final droplet size of the single and double emulsions produced by premix membrane emulsification with SPG membranes, compared to the ones obtained using whey protein alone. Since favorable interaction is formed between both components, they are also less likely to interact with the membrane surface. When using these complexes, an increase in viscosity of the continuous phase will occur and that will increase the pressure needed for premix emulsification to take place, which is as expected.

In summary, it can be said that combinations of construction materials and emulsion components that work well in microfluidic devices are in general also successful for membrane emulsification. We expect that this similarity is due to favorable wetting conditions, that facilitate droplet release through prevention of the to-be-dispersed phase sticking to the wall. How this can be translated to process design is presented in the next section.

### 3.4. Process Guide Lines

**Figure 6:**
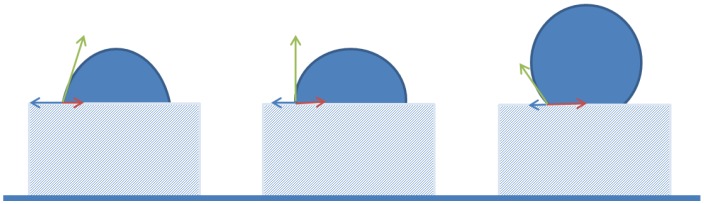
Schematic illustration of the effect of wettability changes that occur in the continuous phase only, going from a weak interaction between continuous phase and solid material (high contact angle for the continuous phase, and high surface free energy that is illustrated in the length of the blue arrow) to increasingly more favorable interactions, which lead to lower contact angles, and ultimately in a change in polarity on the left, with a contact angle < 90° for the continuous phase.

The summary above sounds very simple, but how to achieve these conditions, or how to predict which conditions will occur beforehand? The core to the answer can be found in the contact angle, as is illustrated in Figure 6. The contact angle is a resultant of three surface free energy terms, one between droplet and surface (red), one between continuous phase and surface (blue) and one between droplet and liquid (green). If the arrow is small, this indicates a low surface free energy, which is indicative of compatibility between the two phases. In Figure 6, the surface free energy between continuous phase and surface is stepwise decreased while keeping the other two constant, and this results in a systematic increase in droplet contact angle, thereby going from a droplet to a continuous phase wetted surface. From this it is clear that wettability is very sensitive to changes in surface interactions.

In practice, not just one of the surface free energies will change, mostly more would change and sometimes even all. If the surface gets different properties this would influence both the interaction with the continuous phase and with the droplet phase. If the liquid/liquid interfacial tension decreases as a result of the presence of a surface active component, this mostly also implies that the component will adhere to the solid interface, and changes the surface free energies. What the resulting contact angle will be is hard to predict beforehand, especially when the liquid/liquid interfacial tension is changing very rapidly, as happens during high throughput emulsification processes.

Ideally the wettability for both liquid phase does not change in the presence of the surface active component that is needed to make and stabilize the emulsion, and this implies that the surface active component should be kept from adsorbing to the surface, or that the component forms a layer that does not change in time (so cannot be replaced by any of the phases). It has been demonstrated to be feasible to prevent components from adsorbing, through tailor made modifications, also for construction materials that are used for microfluidic devices (e.g., [23,95]; however, this is very specialized work. It seems to be more realistic to tailor the surface differently, as, e.g., demonstrated by Sahin and Schroën [5] who used a layer of pre-adsorbed protein that cannot be replaced by the oils of choice, and in that way create constant surface properties. Obviously, this layer should be strongly bound and not replaced by either of the liquid phases (e.g., sunflower oil that replaced SDS), or by the emulsifier (see also outlook section).

To check wettability changes, contact angle measurement may be used but for this a specific cell would need to be applied that allows measurement of the contact angle in a liquid system (not against air). The measurement should be done in the absence and presence of surface active components, and steady values should be found. If this is not the case, this is a clear indication of interaction of one of the liquids with the solid surface, or that changes induced by the surface active components take place. These effects become even more important when trying to scale-up microfluidic devices, or using membrane emulsification at large scale [23,32].

As illustrated in Figure 6, not just the solid surface is important, also the liquid/liquid interface, and more specifically that value of the interfacial tension given a certain droplet formation time. The surface active components will be transported and diffuse toward the newly formed interface, thereby decreasing the interfacial tension (e.g., [92]). The actual value of the interfacial tension is highly time dependent, and will range between the value of a completely empty interface (fast droplet formation), and that at equilibrium with the bulk solution (slow droplet formation), and it is not trivial to access the values for the fast droplet formation times that are used in emulsification devices. Starting from a microfluidic Y-junctions, a method that allows estimation of interfacial tension at even sub-millisecond range, has been developed [96,97]. In follow-up research in our labs, other surface active components have also been tested, and it is clear that these are elaborate measurements to carry out, although steady progress is made.

### 3.5. Outlook

The approach described in the previous section has been shown to work for simple emulsions made by direct and premix emulsification. When more complex emulsion-based products are made, such as double emulsions or capsules, all surface active components can be tested as described above. The one with the strongest surface interaction while not changing the wettability of the continuous phase should be chosen to modify the surface. Component evaluation may become a rather impossible task when using mixtures, on the other hand, the natural competition between the surface active components will lead to preferential adsorption of one, and that would also be the one that needs to be tested on replacement by the two liquids. So in effect it is expected that the contact angle test could still be of use, but now it would not be possible to pinpoint the observed effects to a specific component.

Membrane emulsification was also demonstrated to work in the preparation of many solid and hollow particles [98], for a good review please read [54]. Also here the same rules apply as mentioned before, and in case of rapid solidification, also interactions of the solvent (that is needed to be able to work with polymers), with all available interfaces need to be considered. Away from solidification occurring when still in the membrane or microfluidic device, the wettability changes that are related to mass transfer of the solvent to the continuous phase can induce drastic wettability changes as we observed in unpublished work done with the systems described by Sawalha *et al*. [98].

In conclusion, wettability changes occur and can destroy emulsification processes when carried out with microstructured devices such as membranes and microfluidics. This paper will help guide researchers in finding the optimal combination of construction material and the components that can be used safely in the emulsion formulation. Obviously, this would also need to be combined with other process requirements to be successful, but we find that these considerations should be the core of any new process design.

## Figures and Tables

**Table 1:** Maximum productivity of different oil-emulsifier combinations [5].

Emulsifier	Hexadecane			Sunflower Oil		
	Pressure Range (mbar)	@ Maximum Pressure		Pressure Range (mbar)	@ Maximum Pressure	
		Diameter (µm)	Frequency/500 µm Plateau Width (Hz)		Diameter (µm)	Frequency/500 µm Plateau Width (Hz)
SDS	60–115	12	160	40–60	16	12
SDS/WPI ^a^				40–100	12	67
Tween 20	90–210	12	200	60–120	10	50
WPI	170–450	12	1221	125–220	10	28
α-lac	150–470	14	5500	130–210	10	33
β-lac	190–470	13	1073	130–210	10	28
BSA pH 7	190–400	14	150	130–200	10	8
BSA @ pI	220–440	24	526	150–260	26	31
BSA pH 3	190–450	14	1255	Polydisperse		

^a^ Prior to SDS-oil experiment, the channels were modified through protein adsorption.

**Table 2:** Interfacial tension between sunflower oil and water at room temperature for different emulsifier systems used for premix membrane emulsification with nitrocellulose mixed ester (MCE) and PES membranes and final droplet size after five emulsification cycles. Emulsification with Tween 20 or Tween 20 + BSA performed at 500 kPa; emulsification with BSA performed at 900 kPa [91].

Emulsifier	Equilibrium Interfacial Tension (mN/m)	*d*_3,2_ (μm) MCE Membrane	*d*_3,2_ (μm) PES Membrane
Tween 20 (2%)	2.87	1.05	0.67
BSA (1%)	5.71	4.97	3.99
BSA (5%)	3.50	2.57	2.74
Tween 20 (2%) + BSA (1%)	0.74	1.09	0.54
Tween 20 (2%) + BSA (5%)	0.49	1.15	0.53
